# An explainable GeoAI approach for the multimodal analysis of urban human dynamics: a case study for the COVID-19 pandemic in Rio de Janeiro

**DOI:** 10.1007/s43762-025-00172-2

**Published:** 2025-03-03

**Authors:** David Hanny, Dorian Arifi, Steffen Knoblauch, Bernd Resch, Sven Lautenbach, Alexander Zipf, Antonio Augusto de Aragão Rocha

**Affiliations:** 1 Interdisciplinary Transformation University Austria, Altenberger Straße 66c, Linz, 4040 Upper Austria Austria; 2 Department of Geoinformatics, University of Salzburg, Schillerstraße 30, Salzburg, 5020 Salzburg Austria; 3 GIScience Chair, Heidelberg University, Im Neuenheimer Feld 368, Heidelberg, 69120 Baden-Wuerttemberg Germany; 4 Interdisciplinary Center of Scientific Computing, Heidelberg University, Im Neuenheimer Feld 205, Heidelberg, 69120 Baden-Wuerttemberg Germany; 5 Heidelberg Institute for Geoinformation Technology, Schloss-Wolfsbrunnenweg 33, Heidelberg, 69118 Baden-Wuerttemberg Germany; 6 Institute of Computing, Fluminense Federal University, Av. Gal. Milton Tavares de Souza, Niterói, 24210-310 Rio de Janeiro Brazil; 7 Center for Geographic Analysis, Harvard University, 1737 Cambridge Street, Cambridge, 02138 MA USA

**Keywords:** GeoAI, Social media, Mobility, Epidemiology, Time series analysis, Feature selection

## Abstract

The recent COVID-19 pandemic has underscored the need for effective public health interventions during infectious disease outbreaks. Understanding the spatiotemporal dynamics of urban human behaviour is essential for such responses. Crowd-sourced geo-data can be a valuable data source for this understanding. However, previous research often struggles with the complexity and heterogeneity of such data, facing challenges in the utilisation of multiple modalities and explainability. To address these challenges, we present a novel approach to identify and rank multimodal time series features derived from mobile phone and geo-social media data based on their association with COVID-19 infection rates in the municipality of Rio de Janeiro. Our analysis spans from April 6, 2020, to August 31, 2021, and integrates 59 time series features. We introduce a feature selection algorithm based on Chatterjee’s Xi measure of dependence to identify relevant features on an Área Programática da Saúde (health area) and city-wide level. We then compare the predictive power of the selected features against those identified by traditional feature selection methods. Additionally, we contextualise this information by correlating dependence scores and model error with 15 socio-demographic variables such as ethnic distribution and social development. Our results show that social media activity related to COVID-19, tourism and leisure activities was associated most strongly with infection rates, indicated by high dependence scores up to 0.88. Mobility data consistently yielded low to intermediate dependence scores, with the maximum being 0.47. Our feature selection approach resulted in better or equivalent model performance when compared to traditional feature selection methods. At the health-area level, local feature selection generally yielded better model performance compared to city-wide feature selection. Finally, we observed that socio-demographic factors such as the proportion of the Indigenous population or social development correlated with the dependence scores of both mobility data and health- or leisure-related semantic topics on social media. Our findings demonstrate the value of integrating localised multimodal features in city-level epidemiological analysis and offer a method for effectively identifying them. In the broader context of GeoAI, our approach provides a framework for identifying and ranking relevant spatiotemporal features, allowing for concrete insights prior to model building, and enabling more transparency when making predictions.

## Introduction

Understanding urban human dynamics is critical for effective public health interventions during the spread of infectious diseases. The vast amounts of high-dimensional crowd-sourced geo-data available online provide an opportunity to analyse such dynamics from a high-level perspective using Geospatial Artificial Intelligence (GeoAI). Yet, the involved analyses are often complex, facing challenges in interpretability, explainability and model transparency. The complexity of infectious disease spread in geographic space and time further complicates these efforts (Cordes and Castro 2020). Cases and deaths exhibit heterogeneous distributions, and the process of contagion is location-dependent as the virus moves through different regions (Chinazzi et al. 2020). Nearby places are more likely to experience similar infection rates due to increased social and cultural ties, although these connections are not always obvious. Arthur et al. (2017) argued that infectious disease dynamics are a complex system with numerous interacting socio-ecological variables with heterogeneous properties.

The early 2020s, dominated by the Severe Acute Respiratory Syndrome CoronaVirus 2 (SARS-CoV-2) pandemic, exemplify these intricacies. First identified in Wuhan, China, in January 2020, the virus rapidly spread globally, resulting in over seven million deaths by 2024 (WHO 2024). This period, commonly referred to as the COrona VIrus Disease 2019 (COVID-19) pandemic as defined by the World Health Organisation (WHO) in February 2020 (Ciotti et al. 2020), highlights the critical need for advanced geospatial analyses to monitor and mitigate disease spread.

In an effort to minimise the impact of infectious disease spread and improve preparedness, crowd-sourced geospatial data has been used extensively to monitor human behaviour and the environment (Li et al. 2021). For instance, mobile phone data has been utilised to observe human movement patterns (Grantz et al. 2020) and therewith improve the planning of Non-Pharmaceutical Interventions (NPIs). Similarly, it has been demonstrated that sickness-related social media posts can be useful for case count prediction (Kogan et al. 2021; Stolerman et al. 2023). The spread of diseases (Dalvi et al. 2023) and social media posting behaviour (Abitbol and Morales 2021) are also highly influenced by contextual socio-demographic factors.

While numerous spatiotemporal digital data streams have been integrated into a joint analysis in the past (e.g. Stolerman et al. 2023), these previous efforts are restricted to a state or county level and involve no more than six features (Kogan et al. 2021). In addition, the importance of the features for model development has not been considered in this context. We are consequently missing an approach to extract and rank multiple spatiotemporal features from geospatial data according to their association with disease infections. Such a method should explicitly prevent redundancies to eliminate any risk of collinearity or concurvity for subsequent model development.

To address these shortcomings, we introduce a method that is capable of (1) extracting 59 multimodal time series features from mobile phone and social media data (2) ranking and evaluating them on a city and sub-city level and (3) contextualising the features using socio-demographic variables. Our approach combines GeoAI with feature selection to quantify local associations between human mobility, social media posting behaviour and viral infections. It can be used for the model-free assessment of features and allows for the training of transparent prediction models using only the most efficient subset of features, providing a basis for more informed model development in GeoAI.

We chose the municipality of Rio de Janeiro in Brazil as a study area due to the availability of comprehensive COVID-19 case data, mobile phone data, geo-social media posts and socio-demographic information in the time frame from April 4, 2020 to August 31, 2021. However, our methodology can be transferred to arbitrary diseases and locations.

Overall, our study aims to answer three research questions:

1. How can associations between mobility patterns from mobile phone data, social media postings and COVID-19 infection rates in the municipality of Rio de Janeiro be identified and ranked?
2. How do these associations vary across the spatial scales Área Programática da Saúde (AP) (health area) and the city as a whole?
3. To what extent is the association strength of these features linked to socio-demographic factors such as population, ethnicity and social development?

Throughout this work, we make the following contributions:

- We present an explainable GeoAI approach that combines multiple geospatial data sources to enhance the understanding of urban dynamics regarding the COVID-19 pandemic.
- Our workflow is capable of computing time series features from mobile phone and social media data to quantify (1) human mobility, (2) postings regarding certain topics and (3) emotional statements over time.
- As part of our method, we propose a semi-supervised Machine Learning (ML) pipeline that combines topic modelling with zero-shot classification to identify COVID-19-related and other semantic topics of posts simultaneously.
- Furthermore, we present a new algorithm for selecting and ranking time series features based on Chatterjee’s Xi measure of non-linear dependence (Chatterjee 2021) using COVID-19 incidence as a target variable.
- Subsequently, we compare the predictive power of the top 10 features selected by our proposed algorithm with features selected by traditional methods and the respective full feature sets at the local AP and city-wide level.
- Finally, we correlate the association strength of features with COVID-19 with local socio-demographic covariates.

## Related work

Given the scope of this paper, related work includes the spatiotemporal analysis of mobile phone and social media data in public health alongside research examining the influence of socio-demographic factors on the spread of infectious diseases and feature selection in epidemiology.

### Analysis of mobile phone data for public health

Alongside household surveys, mobile phone data is an obvious source of information to monitor human behaviour and mobility (Kalleitner et al. 2022). It can be collected quickly and abundantly for large areas, covering the entire population that owns a cell phone. Mobile phone providers constantly collect Call Detail Records (CDRs) which consist of a Global Positioning System (GPS) location and a timestamp with a unique identifier for each network participant (Grantz et al. 2020). However, it is not always easy to get access to the mobile phone data of the different providers.

Based on the attached information, mobile phone data is well-suited for the assessment of human mobility (Buckee et al. 2020). For instance, Pepe et al. (2020) computed aggregated mobility information from (1) Origin-Destination (OD) movements between Italian provinces, (2) the radius of gyration and (3) the average degree of a spatial proximity network for the assessment of NPIs during the 2020 COVID-19 pandemic in Italy. Similarly, Heiler et al. (2020) assessed the effect of NPIs during the 2020 COVID- 19 pandemic in Austria and analysed daily changes in country-wide mobility patterns using mobile phone data. Specifically, they examined mobile phone traffic at various Point of Interests (POIs), trajectories and the cluster structure of the graph structure represented by the OD matrix. Zhou et al. (2020) used aggregated mobile phone records to build a disease transmission model for COVID-19 cases in the city of Shenzen, China. Specifically, they simulated disease spread while varying types of mobility restrictions and their magnitudes. Oliver et al. (2020) likewise argued that mobile phone data can be a critical tool for supporting public health decisions in all phases of a pandemic in the context of COVID-19.

Dengue has also been subject to mobile phone data analysis. Wesolowski et al. (2015) found that mobile phone-based mobility estimates could be used to predict the geographic spread and timing of Dengue epidemics in Pakistan in 2013. Together with climatic information, they generated fine-grained risk maps to improve epidemic preparedness. Mao et al. (2016) proposed a framework for mapping intra-urban disease risk using cellphone tracking data. Taking the Dengue fever during 2013 and 2014 in Shenzen, China as an example, they used hourly tracking records of 5.85 million cellphone users and mosquito activities in a random forest model to estimate the local transmission risk of Dengue fever (Table 1).

**Table 1:** Contribution beyond State of the Art (SotA)

While mobile phone data has been used frequently for assessing mobility in public health and epidemiology, this information has rarely been combined with other data sources (Okmi et al. 2023). In a related context, Stolerman et al. (2023); Kogan et al. (2021) combined mobility information from Apple mobility with social media data to identify future COVID-19 outbreaks on a state and county level in the United States of America (USA). However, the characteristics of Apple mobility data are different from traditional mobile phone data as it is generated by counting the number of requests to Apple Maps for directions in selected locations.	

### Social media analysis for public health

Similarly to information derived from mobile phone data, social media data has been used extensively to study viral outbreaks such as the 2020 COVID-19 pandemic (Tsao et al. 2021), Ebola (Fung et al. 2016) or Dengue (Kannan et al. 2019). Many previous works examine what and how people post content during or regarding the presence of diseases. For instance, Garcia and Berton (2021) applied topic modelling techniques and sentiment analysis to examine COVID-19-related tweets in Brazil and the USA. de Melo and Figueiredo (2021) conducted a likewise study only for Brazil. Lwin et al. (2020) utilised emotion analysis on global Twitter data during the early stages of the COVID-19 outbreak and found that the output can be useful for decision support to maintain the mental well-being of the population. Park et al. (2020) investigated how COVID-19-related issues have circulated on Twitter using network analysis and applied content analysis to the most shared news sources. They found that most of the news on Twitter was non-medical and that social media analytics can assist decision-making processes in public health. Shahid et al. (2020) analysed 28,688 tweets regarding the Dengue epidemic between 2010 and 2019 in Bangladesh and found several insights helpful for public health officials such as irregularities in Dengue diagnosis and treatment or shortage of blood supply for Rh-negative blood groups. Sharma et al. (2017) examined Facebook as an information source for the Zika virus, starting in 2016. They found that misleading posts were far more popular than posts with accurate public health information.

Social media posts might also be used to predict infection counts as (Shen et al. 2020) demonstrated in a study using around 15 million posts from the Chinese social media platform Weibo. They trained a machine learning classifier to identify sickness-related posts and then estimated the Granger causality of social media posts on daily COVID-19 case counts. The method could predict infections up to 14 days ahead of official statistics. Lucas et al. (2023) presented a model for forecasting COVID-19 incidence at a USA county level using hand-engineered spatial features from Facebook movement and connectedness datasets along with the weekly number of positive cases. The model outperformed the state of the art at the time. Vahedi et al. (2021) conducted a similar study using human mobility and connectivity measures derived from mobile phones and Facebook where they also managed to effectively predict cases of COVID-19 on a county level. Chew et al. (2021) developed a hybrid deep-learning model that was coupled with Twitter data to predict cases of COVID-19 which outperformed traditional time-series models. Ramadona et al. (2019) used human mobility data from Twitter to estimate the importation pressure of Dengue in local neighbourhoods in Indonesia between 2016 and 2018. Their method used a mobility-weighted incidence score which could predict Dengue transmission risk at a three-month lead time (Table 2).

**Table 2:** Contribution beyond SotA

Overall, previous studies were primarily concerned with content analysis and posting behaviour. The few studies that associate social media posts with infection rates are mostly confined to specific regions and use simple keyword filters. In our study, we take on a more comprehensive approach using a semi-supervised ML approach. Furthermore, we combine semantic and emotional information derived from social media which has not been done before in the context of infectious disease analysis.	

### Socio-demographic factors and the spread of viral infections

Socio-demographic factors play a significant role in the spread of viral infections, albeit the findings are often confined to small study areas. For this reason, this section focuses most on previous studies conducted in Brazil. On a country level, Raymundo et al. (2021) found that the Gini index for social inequality was positively correlated with COVID-19. Rafael et al. (2020) analysed the association between per capita income and COVID-19 incidence for the municipality of Rio de Janeiro and found higher incidence rates in regions with high income, suggesting that access to testing occurred unequally in Brazil. Dalvi et al. (2023) investigated socio-demographic and environmental risk factors for the occurrence of Zika, Dengue and Chikungunya among adolescents in the municipalities of Rio de Janeiro and Fortaleza, Brazil between 2015 and 2019. Generally, the risk of contracting one of the Aedes-borne arboviral infections was lower among those living in better socioeconomic conditions. It was also lower among adolescents who attended school in the afternoon rather than the morning. In a similar study, Mocelin et al. (2020) examined the reported cases of Zika virus and congenital Zika syndrome in Espírito Santo, Brazil and related those results to socio-demographic indicators. They found that the Zika virus epidemic primarily affected predominantly non-white women of childbearing age who had completed high school. The case is similar for Chikungunya. Clipes et al. (2024) analysed infection cases in Espírito Santo state, Brazil between 2018 and 2020. Their statistical analysis demonstrated a significant association between race and Chikungunya incidence, though it differed throughout the three years. Similarly, an association between education and incidence was only found in 2020 (Table 3).

**Table 3:** Contribution beyond SotA

So far, studies examining socio-demographic factors and the spread of viral infections primarily focused on direct associations with infectious disease cases. Mobility patterns or social media posting behaviour have not been included in such analyses. In this paper, we put associations between mobility, social media posting behaviour and viral infections into a local socio-demographic context to better understand social dynamics during COVID-19.	

### Feature importance in epidemiology

Techniques from eXplainable Artificial Intelligence (XAI) have recently been popularised to better understand the working principles of black-box ML models. They allow the user to explain the rationale behind predictions (Abdel-Karim et al. 2021). Often, this is achieved through feature importance scores, indicating the predictive power of different input features. In epidemiology, this knowledge can be used to assess human dynamics and associations during outbreaks of viral infections.

In particular, Local Interpretable Model-agnostic Explanations (LIME) (Ribeiro et al. 2016) and SHapley Additive exPlanations (SHAP) (Lundberg and Lee 2017) have been used extensively to study the importance of contextual features for predicting infection rates. Aleixo et al. (2022), for instance used SHAP together with a gradient boosting decision tree model to achieve explainable predictions of Dengue outbreaks in the municipality of Rio de Janeiro. Paul et al. (2021) examined the spread of COVID-19 in the USA and its correlation with socioeconomic metrics and population density. To achieve this, they performed a regression using an ensemble of boosted decision trees and calculated Shapley values to understand the importance of variables. It was found that infections spread asymmetrically in urban and rural areas and primarily affected counties where a large fraction of the population is non-white. Similarly, Banerjee et al. (2022) studied the causal connections between socioeconomic factors and COVID-19 infection in the USA using three different causal orderings. Based on those, causal Shapley values (Heskes et al. 2020) were calculated. They found that socioeconomic metrics such as the fraction of the non-white population showed a significant causal connection with infection rates while population density was only partially connected. Shi et al. (2023) used both SHAP and LIME to compare feature importance for the prediction of COVID-19 mortality using eXtreme Gradient Boosting (XGBoost). Their model revealed that Medicare financial class, older age and gender had a high impact on the mortality prediction. The local interpretation of LIME did not show significant differences in feature importance when compared to SHAP. Lohaj et al. (2023) examined nine prognostic classification models concerning the need for an intensive care unit for COVID-19 patients in Mexico. They then interpreted the models using the learned coefficients, SHAP and LIME and found that those explainability methods identified the same attributes as risk factors as medical experts. Lastly, Attai et al. (2023) used XGBoost to classify febrile diseases’ co-morbidities in pregnant women and children. With the help of SHAP, they then interpreted their model. Upper and lower respiratory tract infections and malaria were identified as the predominant disease co-morbidities in children. For pregnant women, the highest-ranking disease co-morbidities were upper and lower urinary tract infections and malaria (Table 4).

**Table 4:** Contribution beyond SotA

Feature importance scores extracted using XAI address the predictions of already trained models. However, there is little work on the justifiability and explainability of data pre-processing. Feature selection is one of the few methods that allow for an explainable model later on. As (Zacharias et al. (2022), p. 2160) pointed out, neglecting the importance of explainable pre-processing “inevitably leads to insufficient explainability and justifiability of the ML-based system as a whole”. Therefore, we present a method for spatially explicit feature selection which also yields the most important features but does not require any model training.	

## Material and methods

This section describes the data used in our study, continues with data processing, presents our feature selection algorithm and ends with feature evaluation and contextualisation. In our methodology, we

1. designed time series features from mobile phone data, geo-referenced social media posts and COVID-19 case data.
2. applied our feature selection algorithm based on dependence scores computed using Chatterjee’s Xi (2021), which we refer to as a “dependence score” throughout the paper.
3. evaluated the predictive power of the features selected at both the local health area and city-wide scale using a gradient boosting decision tree model, comparing them to those identified by existing feature selection methods.
4. correlated local dependence scores and prediction quality with 15 socio-demographic variables while using Spearman’s rho.

**Fig. 1:**
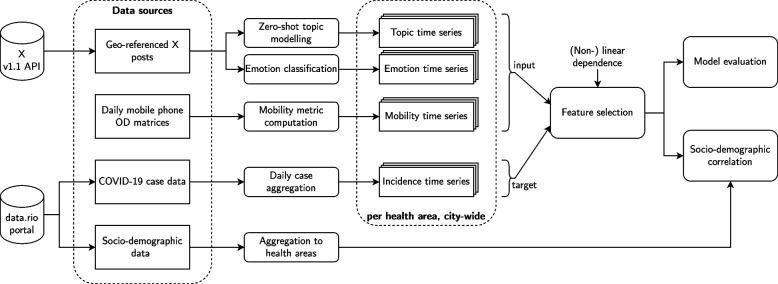
Overview of the methodology presented in this study

A brief summary of our methodological workflow is presented in Fig. 1. Its individual building parts are described in detail below.

### Data

**Fig. 2:**
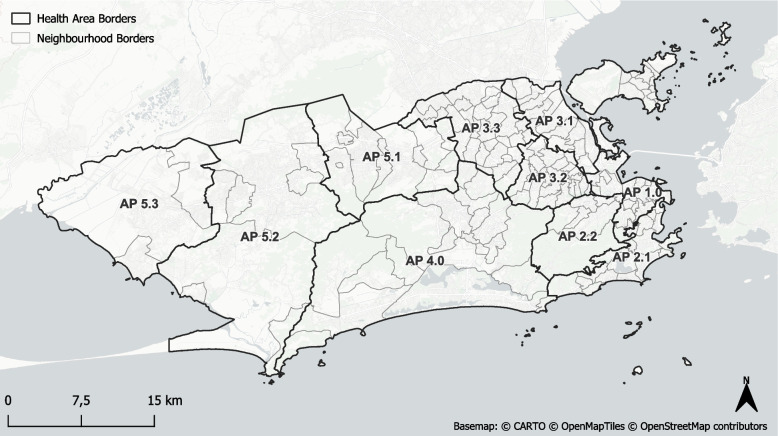
Borders of the health areas (APs) and neighbourhoods of the municipality of Rio de Janeiro as derived from the data.rio portal

Our data sources consisted of (1) COVID-19 case data, (2) mobile phone data in the form of OD matrices, (3) social media posts derived from X, and (4) socio-demographic data from the City Council of Rio de Janeiro. The municipality of Rio de Janeiro is officially divided into 10 APs which we will refer to as *health areas*. The borders are depicted in Fig. 2. Each health area can be broken down hierarchically into neighbourhoods. However, some neighbourhoods are very small and presented only few or no local cases of COVID-19, social media posts or mobility. Therefore, we decided to focus on a health area level in our analysis.

#### COVID-19 case data

Data of individual confirmed cases of COVID-19 in the municipality of Rio de Janeiro was downloaded from the city’s data portal data.rio. The data reflects the database of the Brazilian Ministry of Health. A confirmed case is determined by a conclusive laboratory confirmation for SARS-CoV-2, regardless of signs or symptoms (Ministério da Saúde 2020). Each confirmed case comes with the notification date and a reference to the neighbourhood and health area where the patient stayed. It also contains information on whether the patient died or recovered including the date of evolution. In total, we obtained COVID-19 case data for the entirety of 2020 and 2021. The reliability of this data, however, is limited by the generally low reporting rates for COVID-19 in Brazil. do Prado et al. (2020) estimated a reporting rate of only 7.2% within the state Rio de Janeiro in 2020.

#### Mobile phone data

To model human movement patterns, we obtained daily origin-destination OD matrices using anonymised CDRs provided by a major Brazilian telecommunications company. This raw dataset comprised individual antenna connections from approximately three million users, representing an estimated 45% of the population of the municipality of Rio de Janeiro. The data had a temporal resolution of five minutes, capturing user connections to 1,359 antennas distributed across 163 neighbourhoods (bairros). A connection was recorded when a user sent a text message, used mobile internet data, or made a call. A mobile phone user typically connects to the nearest antenna which serves as a proxy for the user’s location at that time.

In this study, we first generated collective OD matrices for Voronoi tessellations, delineated by the locations of antennas, based on the temporal sequences of individual antenna connections from 06-04-2020 to 31-08-2021. To align this data with our spatial scale of interest, where COVID-19 infection rates and socio-demographic statistics were also collected, the OD matrices at the antenna scale (1,250 x 1,250 cells) were subsequently converted to neighbourhood-level (163 x 163 cells) using methods described by Fabrikant (2019). A more detailed description of the used methodology is provided in Knoblauch et al. (2024).

#### Social media data

To understand human behaviour and reactions during the COVID-19 outbreak in the municipality of Rio de Janeiro, we collected 6,490,355 geo-referenced posts from the microblogging platform X (formerly known as Twitter) using the former v1.1 Application Programming Interface (API). The data was filtered only to include posts with a geo-reference within the city borders of the municipality of Rio de Janeiro and a posting time between 06-04-2020 and 31-08-2021. Each post contains various attributes: text, a timestamp and a geo-reference in the form of either a lat/lon point coordinate or a bounding box polygon.

#### Socio-demographic information

We obtained socio-demographic information for each health area from the city’s data portal data.rio. The features we collected include population, the number of households, population density, average household size, sex ratio (males/females), average age, the fraction of white/black/mixed/Asian/Indigenous population, literacy rate, school enrolment rate for children aged between 7 and 14, Social Development Index (SDI), and average household income in Brazilian Real (R$). Most socio-demographic features could only be retrieved as individual tables that had to be merged using a semi-automatic string-matching technique. If data was only available on a neighbourhood level, we aggregated it to a health area level using the sum or the population-weighted mean.

### Time series creation

A major part of our study consisted of engineering time series features that could be associated with COVID-19 infections. To achieve that, we designed and computed the following temporal features on both a local health area level and city-wide:

1. daily 14-day incidence values for COVID-19 per 100,000 inhabitants,
2. 12 and 2 mobility time series derived from mobile phone data respectively,
3. 39 time series capturing the number of daily posts regarding different semantic topics,
4. 8 time series regarding the emotions associated with posts.

#### COVID-19 incidence

To obtain an indicator of COVID-19 incidence within each health area, we computed the local 14-day incidence per 100,000 inhabitants as depicted in Eq. 1 (WD 2021). The choice of the 14-day window was based on previous studies by Linton et al. (2020) who suggested an incubation period of 2–14 days with 95% confidence and Lauer et al. (2020) who estimated that 99% of people exposed to COVID-19 will develop symptoms within 14 days after exposure to COVID-19. Therefore, the 14-day window effectively captures the majority of ongoing infections. In addition to health-area indicators, we also calculated the incidence on a city-wide scale by summing up the population of all health areas.

1 $$\begin{aligned} \text {14-day-incidence-per-100k} = \frac{\# \text{cases in last 14 days}}{\text {population}} \cdot 100,000 \end{aligned}$$

**Fig. 3:**
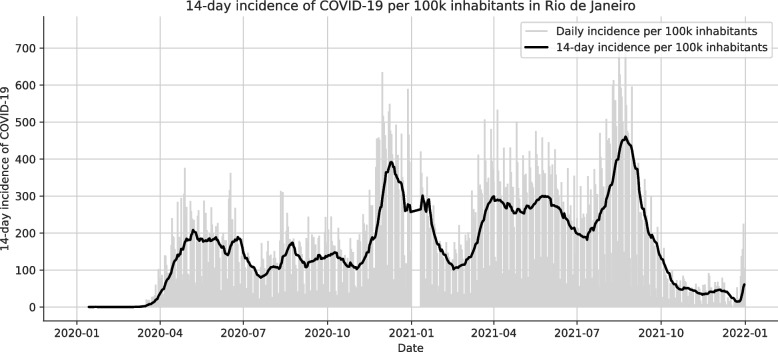
City-wide 14-day incidence of COVID-19 in Rio de Janeiro, computed using Eq. 1

Figure 3 depicts the city-wide 14-day incidence of COVID-19 in Rio de Janeiro between January 2020 and December 2021 visually. There is a small gap in the data after the start of the new year, potentially due to reporting delays. Such delays could not be confirmed formally, however. Additionally, two sharp waves are visible, one starting around November 2020 and one starting in July 2021.

#### Mobility

The daily OD matrices for neighbourhoods derived from mobile phone information were used to calculate several local mobility metrics. We denote the set $\{\textbf{OD}^{(1)}, \textbf{OD}^{(2)}, \dots , \textbf{OD}^{(N)}\}$ as the collection of all matrices where *N* is the total number of days covered. $\textbf{OD}^{(d)}_{k,l}$ denotes the number of people traced with mobile phones moving from neighbourhood *k* to neighbourhood *l* on day $d \in \{1, \dots N\}$ where $k,l \in \{1, \dots 163\}$ are neighbourhood indices. By aggregating neighbourhoods to health areas, we then quantified mobility as follows for each health area $A_j, j\in \{1, \dots , 10\}$.

- $\text {in}_{j,d}$: The number of incoming persons from other health areas $A_i, i \ne j$ on day *d*.
- $\text {out}_{j,d}$: The number of outgoing persons to other health areas $A_i, i \ne j$ on day *d*.
- $\text {internal}_{j,d}$: The number of persons moving between neighbourhoods within health area $A_j$ on day *d*.
- $\text {total}_{j,d}$: The total mobility within health area $A_j$ calculated as the sum of incoming, outgoing and internally moving persons.

To additionally obtain relative mobility statistics, we normalised each of these absolute mobility metrics using the city-wide mobility $\text {total}_{*,d} = \sum \sum _{k \ne l} \textbf{OD}_{k,l}^{(d)}$ which described the total number of persons moving within the city on day *d*. Table 5 summarises all of the mentioned mobility metrics.

**Table 5:** Summary of mobility metrics for each health area $A_j$ and day *d* computed from the daily OD matrices

Absolute mobility	Relative mobility
$\text {in}_{j,d} = \sum \sum _{k \in A_j, l \notin A_j} \textbf{OD}_{l,k}^{(d)}$	$\text {in}_{j,d}^{\text {(norm)}} = \frac{\text {in}_{j,d}}{\text {total}_{*,d}}$
$\text {out}_{j,d} = \sum \sum _{k \in A_j, l \notin A_j} \textbf{OD}_{k,l}^{(d)}$	$\text {out}_{j,d}^{\text {(norm)}} = \frac{\text {out}_{j,d}}{\text {total}_{*,d}}$
$\text {internal}_{j,d} = \sum \sum _{k,l \in A_j, k \ne l} \textbf{OD}_{k,l}^{(d)}$	$\text {internal}_{j,d}^{\text {(norm)}} = \frac{\text {internal}_{j,d}}{\text {total}_{*,d}}$
$\text {total}_{j,d} = \text {in}_{j,d} + \text {out}_{j,d} + \text {internal}_{j,d}$	$m_{j,d}^{\text {(norm)}} = \frac{\text {total}_{j,d}}{\text {total}_{*,d}}$

Subsequently, we used the granular knowledge about movement in between neighbourhoods to calculate the average travel distance. For an OD matrix entry $\textbf{OD}_{k,l}^{(d)}$, the associated travel distance $d_{k,l}$ is the distance from the centroid of neighbourhood *k* to the centroid of neighbourhood *l* in metres. We used this information to quantify the average travel distance for incoming, outgoing, internal and total mobility by calculating the weighted arithmetic mean where the weights are the relative fractions of persons moving. The calculations are depicted in Table 6.

**Table 6:** Formulas of the average travel distance metrics for each health area $A_j$ and day *d* computed from the daily OD matrices

Average travel distance	
$$d^{\text {(in)}}_{j,d} = \sum \sum _{k \in A_j, l \notin A_j} \frac{\textbf{OD}_{l,k}^{(d)}}{\text {in}_{j,d}} \cdot d_{l,k}$$	
$$d^{\text {(out)}}_{j,d} = \sum \sum _{k \in A_j, l \notin A_j} \frac{\textbf{OD}_{k,l}^{(d)}}{\text {out}_{j,d}} \cdot d_{k,l}$$	
$$d^{\text {(internal)}}_{j,d} = \sum \sum _{k,l \in A_j, k\ne l} \frac{\textbf{OD}_{k,l}^{(d)}}{\text {internal}_{j,d}} \cdot d_{k,l}$$	
$$d^{(\text {total})}_{j,d} = \sum \sum _{k \in A_j \vee l \in A_j, k\ne l} \frac{\textbf{OD}_{k,l}^{(d)}}{\text {total}_{j,d}} \cdot d_{k,l}$$	

Overall, this resulted in 12 time series derived from mobility data for each health area. Last, we smoothed them using a 14-day moving average window to reduce fluctuations due to weekly patterns, weather, holidays or special events. Noland (2021) found that changes in mobility are reflected in the effective reproduction rate of COVID-19 within 7 to 14 days. A 14-day window can capture these changes effectively.

Analogously, we computed the city-wide mobility time series for the comparison across spatial scales. This reduced to merely the internal movement and average internal travel distance since the number of incoming and outgoing persons was always zero and normalisation as described above was not possible. Consequently, we obtained 2 time series capturing the mobility city-wide.

#### Social media

**Fig. 4:**
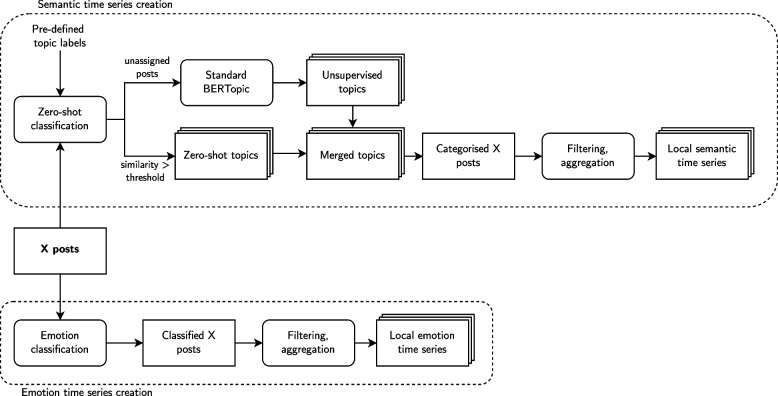
Visual overview of time series creation from social media posts

To extract quantitative information about posting behaviour on social media, we took a two-fold approach: First, we used a semi-supervised topic modelling approach combined with zero-shot classification to categorise posts into semantic topics. Subsequently, we constructed posting frequency time series for each topic. Second, we performed multilingual emotion classification and created similar time series for the emotional association of posts. A visual overview of our social media processing workflow is depicted in Fig. 4.

##### Semantics

To quantify how many posts were sent each day for the different semantic topics, we utilised a joint approach that combines zero-shot classification with unsupervised topic modelling based on BERTopic (Grootendorst 2022). Topic modelling is the unsupervised task of categorising documents into multiple collections that are coherent semantically. There are generally two approaches to it: Traditional bag-of-words based methods such as Latent Dirichlet Allocation (LDA) (Blei et al. 2003) which infer topics based on word frequencies and clustering-of-embeddings-based methods like BERTopic (Grootendorst 2022). BERTopic has specifically been shown to consistently outperform traditional approaches in topic coherence and diversity which is why we used it for our study (Egger and Yu 2022; de Groot et al. 2022).

Since purely unsupervised topic modelling did not capture all potentially useful topics regarding COVID-19 by default, we integrated prior knowledge from experts into the procedure. Previous studies conducted by Shen et al. (2020); Stolerman et al. (2023) suggest that the quantity of symptom- and COVID-19-related posts on social media holds predictive power for infection cases. Additionally, high rates of tourism and mobility tend to correlate with incidence rates (Zhou et al. 2020) and are also represented on social media (Zeng and Gerritsen 2014). For this reason, we decided to take on a guided zero-shot topic modelling approach based on BERTopic that works as follows:

1. First, pre-defined zero-shot topics are defined through labels. Each label provides a short description of each topic, e.g. *symptoms of sickness like fever, cough and nausea*. Posts mentioning symptoms of sickness should then be assigned to this topic. This way, prior knowledge can be integrated into the topic modelling procedure.
2. Next, the labels are embedded in a semantic space using the same embedding model that BERTopic utilises later on. In our case, we used *distiluse-base-multilingual-cased-v1* (Yang et al. 2020).
3. Documents, for which the embedding has cosine similarity $>t$ for at least one of the labels, are assigned to the pre-defined topic with maximum similarity. In our study, we set $t=0.3$ as this setting achieved the highest zero-shot classification accuracy (0.91) on a sample of size 32 posts in a grid search within [0, 1] with step size 0.1.
4. All remaining documents that could not be assigned to one of the pre-defined topics are then used as input to a standard BERTopic model.
5. Finally, the zero-shot topics are combined with the remainder topics based on similarity as in the default implementation of Grootendorst (2022).

For the zero-shot topics, we decided to go with the following four labels based on the findings of Stolerman et al. (2023); Shen et al. (2020) who showed that COVID-19-related tweets hold early-warning capabilities for infection outbreaks. We also incorporated a topic about tourism and going out based on the results of Zhou et al. (2020).

- Symptoms of sickness based on the results of Shen et al. (2020): *sintomas do doença, como febre, tosse ou náusea*
- Posts explicitly related to COVID-19 and the pandemic: *coronavírus, pandemia, lockdown devido à COVID* (Stolerman et al. 2023)
- Posts explicitly related to arboviral diseases to filter those out explicitly: *dengue, zika e febre chikungunya, vírus transmitidos por mosquitos*
- Tourism and party since the mobility of people is usually associated with higher incidence rates (Zhou et al. 2020): *turismo, festas e baladas no Rio de Janeiro*

Going further, we pre-processed each of our 6,490,355 posts by replacing user references and links with the standardised tokens “@user” and “http” and removing posts that only contained sequences of “k” (e.g. “kkkkkkk”) which represent laughing, following an approach by Resch et al. (2018).

We then applied the zero-shot BERTopic method in a step-wise manner using the *distiluse-base-multilingual-cased-v1* model for embedding generation, Uniform Manifold Approximation and Projection (UMAP) for dimensionality reduction (McInnes et al. 2020) and Hierarchical Density-Based Spatial Clustering of Applications with Noise (HDBSCAN) for clustering (Campello et al. 2013) as this setup has repeatedly been shown to deliver the highest topic coherence and diversity (Grootendorst 2022; Angelov 2020; Hanny and Resch 2024). The minimum cluster size was set to 5,000 to allow for the creation of reasonable time series in each of the ten health areas of Rio de Janeiro over the 512-day period the data covers. Additionally, we set the total number of topics to 20, where 4 of which were the zero-shot topics, and 16 were the topics computed unsupervised from the remaining documents. This setup yielded the highest topic quality (0.22) measured by the product of coherence diversity (Dieng et al. 2020) in a grid search within the range [10, 25], though all other settings also produced overall quality scores between 0.21 to 0.22. Finally, to achieve a human-readable representation of topics, we used GPT-4o-mini (OpenAI 2024) to compute a summarising topic label of up to eight words based on the topic keywords and a sample of up to 3,000 posts from each cluster.

The final output, detailed in Sect. 8, consisted of 19 topics, where one unsupervised topic was merged with a zero-shot topic. For each topic and health area, we created semantic time series by counting the absolute number of topic-specific posts per day within the respective health area boundaries. To account for variations in daily activity, we normalised these counts by the total number of posts made each day. Additionally, we generated a time series of the total daily posts within each health area. To capture the overall trend and to reduce the impact of outliers, we computed a 21-day moving average for each time series. The 21-day window was chosen based on the findings of Kogan et al. (2021) who showed that increases in COVID-19-related tweets can precede increases in confirmed cases by 2 to 3 weeks. Overall, we obtained 39 time series per health area: two for each topic (absolute and normalised counts) and one for the total number of posts. We also computed the same 39 time series on a city-wide scale by summing up all posts within the city boundaries for each day to compare the associations on a health area level with associations observed city-wide.

##### Emotions

We also computed time series for the emotions present on social media analogously to the semantic time series. To achieve this, we used XLM-EMO, a multilingual emotion classification model by Bianchi et al. (2022) which is based on a multilingual variant of RoBERTa trained on Twitter data (Conneau et al. 2020; Barbieri et al. 2022). It was evaluated on social media data in 19 languages including Portuguese, Spanish and English, for which it consistently achieved a macro-F1 score of 0.85 to 0.9. We used it to categorise each pre-processed social media post into one of the emotions *joy, anger, fear, sadness*.

Subsequently, time series for the daily number of associated posts were built for each emotion in each health area by aggregating the tweets by their geometry and date. As for the semantic time series, we computed both the absolute number of posts per day and the fraction when related to the total number of posts made that day and applied a 21-day moving average. This resulted in 8 emotion time series per health area: two for each emotion (absolute and normalised). Again, we calculated the same 8 emotion time series on a city-wide scale by summing up all posts within the city boundaries for each day to compare associations across the spatial scales *health area* and *city-wide*.

### Feature selection

To extract associations in the data, we designed a feature selection algorithm based on Chatterjee’s Xi measure of dependence (Chatterjee 2021), which is denoted as $\xi _n(\cdot , \cdot )$. The goal was to obtain the top time series features associated most strongly with COVID-19 incidence while simultaneously minimising redundancies. Our algorithm iteratively selects the feature with the highest dependence score (in our case, Chatterjee’s Xi) and subsequently prunes all inter-dependent features. This process is repeated until no more features above a certain threshold are identified or a maximum number of features is reached. Let $\vec f_1, \dots \vec f_n$ be our time series features derived from the previous steps and $\vec t$ the time series holding COVID-19 incidence values. Although we used Chatterjee’s Xi to measure dependence in this study, the algorithm is suitable for any dependence metric. It can be summarised as follows:

1. Conduct an independence test for each pair of time series $(\vec f_i, \vec t)$ and keep only the features $\vec f_i$ for which the null hypothesis of independence can be rejected as candidates.
2. Shift each candidate time series feature $\vec f_i$ by the temporal lag $l \in \{0, \dots , L\}$ in days such that the dependence $\xi _n(\vec f_i, \vec t)$ is maximised.
3. Compute $\xi _n(\vec f_i, \vec t)$ for all shifted candidate features and sort them descendingly.
4. Select the top features using greedy selection followed by candidate pruning:
  - Retrieve the feature $\vec f$ with the highest dependence score $\xi _n(\vec f, \vec t)$ from the candidate list.
  - Return $\vec f$, the dependence score $\xi _n(\vec f, \vec t)$ and the associated optimal temporal lag *l*.
  - Remove $\vec f$ and all features for which $\xi _n(\vec f, \vec f_i)> \beta$ from the candidate list.
  - Repeat the above steps until there is no feature $\vec f_i$ for which $\xi _n(\vec f_i, \vec t)> \alpha$ any more or the specified number of features is reached.

The algorithm not only provides the top features based on a dependence threshold or an absolute number but also returns the associated dependence score and optimal temporal lag in days. It is parametrised by the maximum temporal lag *L* in days, the threshold values $\alpha$, $\beta$ and the maximum number of features.

We used Chatterjee’s Xi (2021) in this context as it provides a simple, intuitive coefficient that is a consistent estimator of dependence taking on a value of 0 if and only if the two input variables are independent and 1 if and only if one is a measurable function of the other. Moreover, it has a simple asymptotic theory which allows for the quick computation of *p* values for testing independence.

The coefficient follows a rank-based formula. Let *X* and *Y* be a pair of random variables with i.i.d. sample pairs $(x_1, y_1), \dots (x_n, y_n)$. Assuming that there are no ties, $\xi _n$ is calculated as follows: First, the data is re-arranged as $(x_{(1)}, y_{(1)}, \dots x_{(n)}, y_{(n)})$ such that $x_{(1)} \le \dots \le x_{(n)}$. We then let $r_i$ be the rank of $y_{(i)}$. Chatterjee’s Xi correlation is then derived from Eq. 2.

2 $$\begin{aligned} \xi _n = 1 - \frac{3 \sum _{i=1}^{n-1} |r_{i+1} - r_i|}{n^2 -1} \end{aligned}$$

We applied the above algorithm to select local time series features for each health area, using the corresponding local mobility, semantic and emotion time series features as inputs $\vec f_1, \dots \vec f_n$ and the local COVID-19 incidence as $\vec t$. The parameters were set to $\alpha = 0.3$, $\beta = 0.5$ and a maximum of 10 features, ensuring that the output remained concise and interpretable. Additionally, we set $L=21$, restricting the range of possible temporal lag values to [0, 21] since only information from the past or present might be used to make predictions. The same configuration was used to obtain time series features at a city-wide level, using the city-wide mobility, semantic and emotion time series features as inputs $\vec f_1, \dots \vec f_n$ and the city-wide COVID-19 incidence as $\vec t$. Consequently, we obtained a list of $\le 10$ triples of the form $(\text {feature name}, \text {dependence score}, \text {optimal temporal lag})$ for each health area and city-wide.

### Model evaluation

Next, we evaluated the quality of the selected features by comparing their predictive power against those returned by traditional feature selection methods and the full feature sets. To this end, we trained separate prediction models for each health area and for the city as a whole based on the following feature selection approaches:

- our feature selection approach at the health area scale, i.e. using health-area-level data as input, which we abbreviate as Local Feature Selection (LFS),
- our feature selection approach at the city scale, i.e. using city-level data as input, which we abbreviate as City-wide Feature Selection (CFS),
- *k*-best feature selection based on Mutual Information (MI), choosing the features with the highest MI regarding the target variable (in our case COVID-19 incidence) (Vergara and Estévez 2014),
- Least Absolute Shrinkage and Selection Operator (LASSO) regression where we conducted a grid search in the search space [0.00001, 500] using step size 10 for the $L_1$ penalty during each fit and then extracted the top *k* features with the highest absolute coefficient values from the best-fitting model (Tibshirani 1996),
- Recursive Feature Elimination (RFE) using a gradient boosting decision tree model with the configuration described below as the estimator. Features were removed recursively in descending order of the model-based importance until only the top *k* features were left (Guyon et al. 2002),
- Feature Ordering by Conditional Independence (FOCI) which is forward stepwise feature selection based on a conditional dependence coefficient inspired by Chatterjee’s Xi (2021) and
- no feature selection, i.e. utilising all 59 available features at the health area scale and all 49 at the city scale.

Each feature selection method was parametrised to return a maximum of 10 features and applied independently to the time series data constructed for each health area and the city as a whole, as described in Sect. 3.3. Consequently, we obtained up to 7 sets of features considered as most suitable for predicting COVID-19 incidence for each administrative unit at each spatial scale. Table 7 provides an overview of the methods applied at each level.

**Table 7:** Overview of the feature selection algorithms evaluated for each spatial scale as part of the model evaluation. LFS denotes our local feature selection approach described in Sect. 3.3 and CFS its city-wide equivalent

Evaluated per health area	Evaluated city-wide
**LFS**	**CFS**
**CFS**	*k*-best MI
*k*-best MI	LASSO
LASSO	RFE
RFE	FOCI
FOCI	(all 49 features)
(all 59 features)	

To assess the predictive power of these feature sets, we performed a separate train/test/validation split for the time series associated with each administrative unit, i.e. each health area and the city as a whole, using 70% of the data for training, 20% for testing, and 10% for hyperparameter validation. In this context, each data point represented a day within the study period. For our proposed LFS and CFS methods, the input time series were shifted by their respective optimal temporal lag prior to training. Subsequently, we trained individual gradient boosting decision tree models for each health area and the city as a whole, utilising each feature set. Each model was configured with the following parameters:

- a maximum of 500 estimators,
- early stopping after 25 rounds (5% of the maximum estimators) without improvement in the validation squared error,
- a maximum tree depth of 3,
- a learning rate of 0.05.

The maximum tree depth and learning rate were obtained through a grid search, where a model was fitted using all input features per health area and the mode of the best parameters across all health areas was selected. The grid search explored the parameter space $\text {max.-tree-depth} \in \{3,5,7\}, \text {learning-rate}\in \{0.05, 0.1, 0.2\}$ and was based on 5-fold cross-validation on the training data. We chose the gradient boosting model because it can capture both linear and non-linear relationships between features, making it well-suited for our data (Friedman 2001). After training, each model was evaluated on its respective test data using the following performance metrics:

1. RMSE which measures the average magnitude of the errors between predicted and actual values, providing insight into the model’s overall prediction accuracy,
2. the standard deviation of the RMSE denoted as $\sigma _{\text {RMSE}}$, indicating the variability of the prediction errors across health areas, therefore offering a sense of consistency regarding the model’s performance,
3. Mean Absolute Error (MAE), providing a similar insight to RMSE while being less sensitive to outliers,
4. the coefficient of determination $R^2$ which indicates the goodness of the model fit using the proportion of the variance in the dependent variable that can be explained by the independent variables,
5. Mean Squared Logarithmic Error (MSLE) which evaluates the mean squared logarithmic errors, emphasising the relative differences between predicted and actual values and
6. Mean Absolute Percentage Error (MAPE) which measures the mean absolute percentage deviation, thus providing a percentage-based accuracy measure.

In summary, we trained and evaluated separate models for each individual health area, using only the time series specific to that area. In addition, we only used city-level time series data for training and evaluation of the features selected on a city-wide scale. To understand how the features selected locally compared to those selected city-wide, we also evaluated how well the city-wide feature set performed when applied to individual health areas. Moreover, we assessed the predictive performance of all 59 and 49 features for each health area and city-wide, respectively.

### Correlation with socio-demographics

Finally, we associated the results of feature selection and model evaluation with the socio-demographic variables described in Sect. 3.1. Specifically, we used the Spearman rank correlation coefficient (Spearman 1904) to identify monotonic relationships between the dependence scores of our time series features, model error and the socio-demographic variables. Our choice to use the traditional Spearman rank correlation was motivated by the ability to clearly interpret the results, capturing consistent trends. To filter out correlations that were not significant, we conducted a hypothesis test with $H_0$ being that the two input samples are uncorrelated and removed all correlations with $p> 0.05$ from the output.

## Results

The results of our analyses are fourfold: (1) the topic identified during our zero-shot topic modelling procedure, (2) the top features associated most strongly with COVID- 19 incidence per health area, (3) the predictive power of those features, and (4) the correlations between feature dependence scores and socio-demographics.

### Zero-shot topic modelling

The output of the zero-shot topic modelling procedure described in Sect. 3.2.3 is depicted in Table 8. The model identified 16 unsupervised topics, one of which was only comprised of outliers. These were then joined with the 4 zero-shot topics. In the process, the zero-shot topic *dengue, zika e febre chikungunya, vírus transmitidos por mosquitos* was merged with an unsupervised topic about vaccines due to the similarity of the keyword representation as described by Grootendorst (2022), suggesting that the posts were not particularly clear about their topic.

**Table 8:** Topics identified by the zero-shot topic modelling procedure based on BERTopic excluding outliers with no clear assignment. To ease the visualisation of our results, we also assigned a short abbreviation to each of the topics

Topic	Size	Label	Abbreviation
0	261,614	Rio de Janeiro Moments and Experiences	RJMoments
1	29,264	COVID-19 Experiences and Reactions in Brazil	COVIDExp
2	19,308	Health Struggles: Pain, Hunger, and Emotion	HealthStruggles
3	14,159	COVID-19 Vaccination Experiences and Reactions	COVIDVax
4	533,277	Casual Conversations and Inside Jokes	CasualJokes
5	148,879	Daily Life and Humor in Rio de Janeiro	RJLife
6	108,958	Brazilian Football Conversations and Reactions	FootballTalk
7	91,619	Humorous Reactions and Emotions in Football Culture	FootballHumor
8	85,874	Expressions of Faith and Peace in Life	FaithPeace
9	47,004	Emotional Expressions and Social Connections	EmoSocial
10	44,460	Reflections on Birthdays and New Beginnings	Birthdays
11	31,753	Celebrating Lives Through Dance and Videos	DanceLife
12	15,716	Discovering Affordable Hotels and Attractions	TravelTips
13	13,670	Casual Conversations and Everyday Desires	CasualTalk
14	13,142	Desire for Beach and Relaxation	BeachLife
15	7,135	Truths and Lies in Social Interactions	TruthLies
16	6,947	Tattoos: Desires, Experiences, and Emotional Connections	TattooLife
17	5,807	Celebrating Love: Princesas and Reis in Bonds	LoveBonds

Further along in this paper, the labels and abbreviations above are used as names for the semantic time series features. We also manually assigned meaningful labels and abbreviations to the other features based on mobility and emotions. Normalised features (e.g. posts in topic *i* relative to the total number of posts in the area) marked with the suffix “-Norm” in abbreviations (e.g. COVIDExpNorm).

### Feature selection

**Fig. 5:**
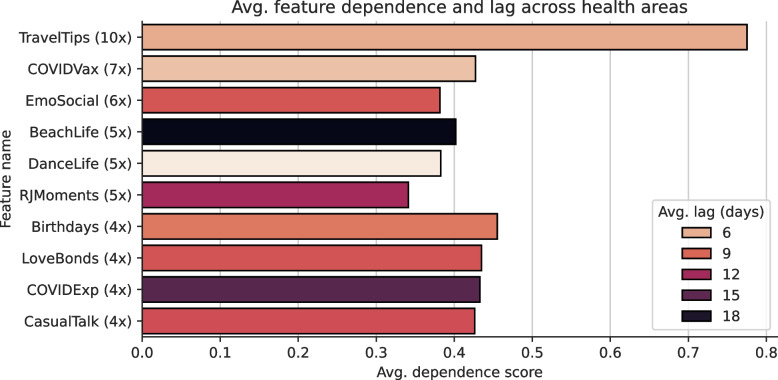
Average dependence score regarding COVID-19 incidence (measured by Chatterjee’s Xi), mean of the optimal temporal lag (indicating the time shift that yielded the highest dependence) and occurrence rate of the 10 features selected most frequently by our algorithm described in Sect. 3.3 on a health area level

**Fig. 6:**
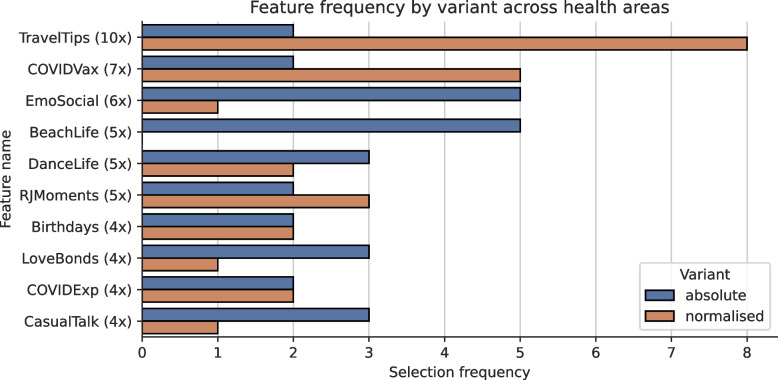
Frequency by variant of the 10 most frequently selected features on a health area level

Across the 10 health areas of the municipality of Rio de Janeiro, our feature selection algorithm returned 5 features for AP 5.2, 8 features for AP 2.1, 9 features for AP 2.2 and 10 for all other APs, resulting in an average of 9.2 features per health area. These selected features demonstrated the strongest association with COVID-19 incidence within their respective health area. Figure 5 depicts the 10 features picked most frequently across all health areas, along with their average dependence score across all health areas and the average optimal temporal lag which indicates the time shift for which the dependence score was highest. To account for the two variants computed for each feature – absolute and normalised – we accumulated both variants for the frequency computation. The absolute variant represents the raw count or value of a feature while the normalised variant adjusts this count relative to the total number of posts or total movement. Information about the ratio is depicted in Fig. 6.

Notably, all frequently selected features were derived from semantic topics on social media. The time series features concerned with *Discovering Affordable Hotels and Attractions* (10 health areas, $\bar{\xi }_n = 0.76$), *COVID-19 Vaccination Experiences and Reactions* (7 health areas, $\bar{\xi }_n = 0.43$) and *Emotional Expressions and Social Connections* (6 health areas, $\bar{\xi }_n = 0.38$) were returned most frequently by our algorithm. Consequently, they were associated strongly with COVID-19 incidence across the majority of the municipality of Rio de Janeiro. Slightly lower-ranked features were the social media topics *Desire for Beach and Relaxation* (5 health areas, $\bar{\xi }_n = 0.40$), *Celebrating Lives Through Dance and Videos* (5 health areas, $\bar{\xi }_n = 0.38$) and *Rio de Janeiro Moments and Experiences* (5 health areas, $\bar{\xi }_n = 0.34$). Notably, the time series concerned with *COVID-19 Experiences and Reactions in Brazil* was returned in only 4 health areas ($\bar{\xi }_n = 0.43$).

The average optimal temporal lag of all features was $\ge 3$ days across all health areas, indicating that the time series were best shifted into the future to yield the highest dependence regarding COVID-19 incidence. Vice versa, this means that COVID-19 incidence was associated most strongly with values that occurred a few days in the past. For *Discovering Affordable Hotels and Attractions*, the average optimal lag was 6 days and for *COVID-19 Vaccination Experiences and Reactions*, the optimum was 5 days. The time series regarding *Emotional Expressions and Social Connections* was best shifted by 9 days and for *Desire for Beach and Relaxation*, the average was 19 days. Overall, these results indicate that a change in incidence was usually preceded by a change of values in the semantic time series.

**Fig. 7:**
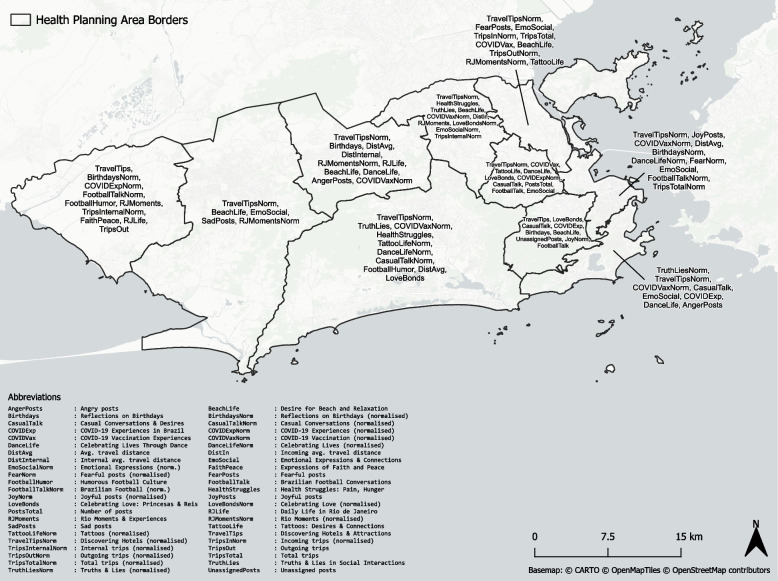
The top locally selected features for each health area in the municipality of Rio de Janeiro, sorted descendingly by dependence score (Chatterjee’s Xi)

Figure 7 visualises the features selected locally per health area, highlighting the spatial heterogeneity in the associations between different features and COVID-19 incidence. Notably, almost all of the features were derived from social media, and within that subset, semantic features were chosen most frequently. This includes the COVID-19-related time series features labelled as *COVID-19 Vaccination Experiences and Reactions* and *COVID-19 Experiences and Reactions in Brazil* with maximum dependence scores of 0.54 and 0.50, respectively. The overall highest dependence score was achieved by the tourism-related time series *Discovering Affordable Hotels and Attractions* which yielded a maximum dependence score of 0.88. The highest dependence score among the emotional time series was achieved by *Fearful posts* which occurred in 2 health areas and achieved a maximum dependence score of 0.58. The highest-scoring mobility feature was the total *Avg. travel distance* which occurred in 3 health areas and achieved a maximum dependence score of 0.47, followed by the normalised *Internal trips* with an occurrence rate of 2 times and a maximum dependence score of 0.37.

**Fig. 8:**
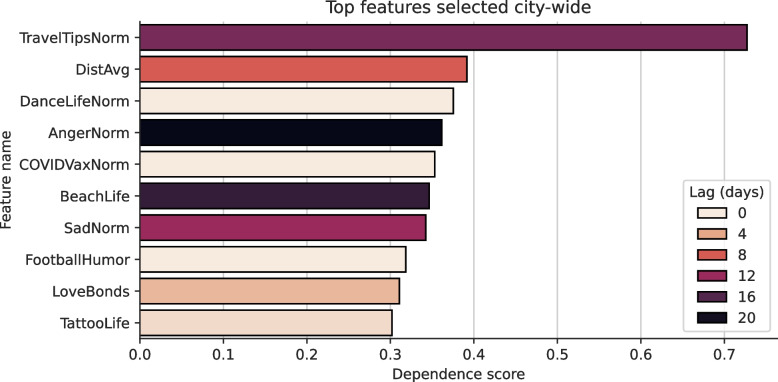
The top 10 features selected city-wide in the municipality of Rio de Janeiro, along with their dependence score (Chatterjee’s Xi) and the optimal temporal lag which indicates the time shift that yielded the highest value. The feature names along the y-axis are abbreviations which are written out fully below the main plot

Zooming out to the city-wide scale, the top 10 globally selected features are depicted in Fig. 8. Again, tourism-, leisure- and COVID-19-related semantic features were ranked highly. The only mobility-related feature present was the *Avg. travel distance* which achieved a dependence score of 0.39. Additionally, the normalised fraction of angry and sad posts achieved dependence scores of 0.36 and 0.34, respectively. The overall trend of the optimal temporal lags was similar to the local case, though slightly more polarised with 5 features yielding an optimal lag of $\le 3$ days and 4 features exhibiting optimal values of $\ge 12$. In contrast to the local case, *Discovering Affordable Hotels and Attractions (normalised)* had an optimal temporal lag of 14 days, while *COVID-19 Vaccination Experiences and Reactions (normalised)* yielded an optimal temporal lag value of 0 days.

### Model evaluation

Table 9 summarises the average performance of the gradient boosting decision tree models trained and evaluated on a health-area level. The features returned by our local feature selection approach (denoted as LFS) yielded an average RMSE of 18.971 cases of COVID-19 per 100,000 inhabitants with a standard deviation of 5.908 which were the best scores among all feature selection methods. The LFS approach also yielded the lowest average MSLE of 0.012. Conversely, the highest average $R^2$ was achieved by RFE, though it outperformed our LFS approach only by a very small margin. The lowest average MAE and MAPE was attained using the features returned by our city-wide selection approach (denoted as CFS). While the full set of 59 features resulted in the best scores across all metrics, our LFS method delivered competitive performance using 10 or fewer features, performing better or on par with traditional feature selection approaches.

**Table 9:** Average local model performance for the features selected by different selection algorithms at a health area level. LFS denotes our local feature selection approach and CFS the city-wide feature selection approach. Additionally, the model performance when all explanatory features were used is depicted. The best scores among the evaluated feature selection methods are marked in bold

Method	RMSE $\downarrow$	$\sigma _{\text {RMSE}}$ $\downarrow$	MAE $\downarrow$	$R^2$ $\uparrow$	MSLE $\downarrow$	MAPE $\downarrow$
**LFS**	**18.971**	**5.908**	13.223	0.958	**0.012**	7.904
**CFS**	19.522	6.112	**12.881**	0.955	0.014	**7.763**
*k*-best MI	19.846	7.909	12.929	0.957	0.016	7.960
LASSO	21.756	9.382	14.484	0.944	0.018	8.916
RFE	19.277	5.977	12.900	**0.959**	0.014	7.882
FOCI	27.380	14.127	18.470	0.904	0.030	11.636
All	14.820	4.883	10.092	0.975	0.009	6.204

**Fig. 9:**
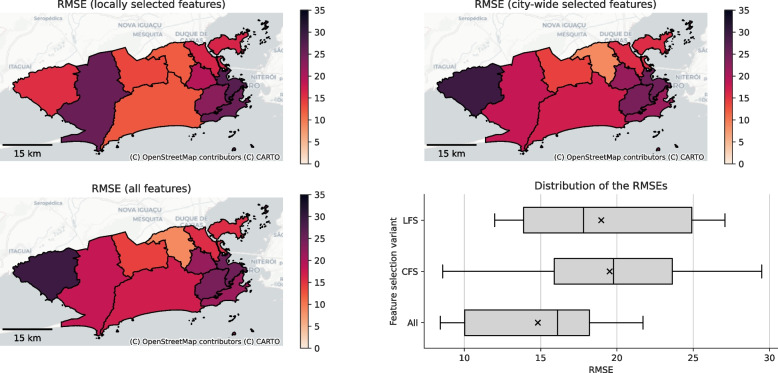
The RMSEs of the prediction model trained using different feature sets and evaluated on the test data. The $\times$ in the boxplot marks the mean. OpenStreetMap has been used for the base maps

Looking at the spatial distribution of errors, Fig. 9 depicts the RMSE per health area for the feature sets returned by our LFS and CFS methods as well as for the full feature set. The RMSE was generally lower in the East of the city for the locally selected features compared to the feature set that was selected at the city level. In the West, the local feature set underperformed for AP 5.2 which can partly be traced back to the fact that only 5 features were selected in this area. In contrast, the city-wide features yielded a higher error for AP 5.3. Notably, APs 5.2 and 5.3 exhibit the lowest and highest overall social vulnerability in the municipality of Rio de Janeiro as shown by Malta and Marques da Costa (2021). We also computed global Moran’s *I* using Queen contiguity weights for the RMSE of all three feature sets. The full set of all 59 features yielded $I=0.23$ with $p=0.068$, indicating a slight tendency towards a spatial clustering of errors, though the *p*-value was $\ge 0.05$. For the locally selected feature, we obtained $I=0.03$ with $p=0.267$ and for the features selected city-wide, the result was $I=0.19$ with $p=0.095$. The overall error ranges in the box plot are lowest for the full feature set. The LFS approach generally performed better than the CFS approach which yielded the largest range of errors.

Table 10 presents the performance of gradient boosting decision tree models trained and evaluated at the city scale. The feature sets derived from our CFS approach yielded competitive performance across several metrics, achieving the lowest average MAPE of 4.768 and the lowest MAE of 8.801. RFE performed best in terms of RMSE, achieving the lowest score of 11.750, and MSLE for which it scored the minimum of 0.004. Nonetheless, the CFS approach maintained robust overall performance, achieving values better than or on par with LASSO, FOCI and *k*-best MI across all metrics. The full set of all 49 city-wide features resulted in consistently strong scores across all error measures, though it was outperformed by RFE in terms of RMSE, indicating a significant amount of noise among the 49 features.

**Table 10:** Average city-wide model performance for the features selected by different selection algorithms. CFS denotes our city-wide feature selection approach. Additionally, the model performance when all explanatory features were used is depicted

Method	RMSE $\downarrow$	MAE $\downarrow$	$R^2$ $\uparrow$	MSLE $\downarrow$	MAPE $\downarrow$
**CFS**	13.757	**8.801**	0.979	0.005	**4.768**
*k*-best MI	13.625	9.815	0.979	0.005	5.336
LASSO	14.378	10.150	0.977	0.006	5.500
RFE	**11.750**	8.810	**0.984**	**0.004**	4.870
FOCI	18.105	10.938	0.963	0.008	5.932
All	13.258	8.865	0.980	0.006	4.979

### Correlation with socio-demographics

**Fig. 10:**
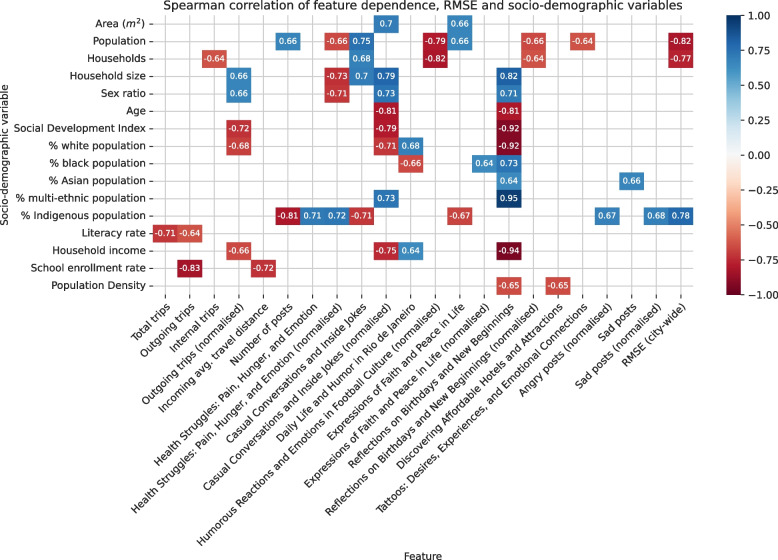
Spearmann’s rank-correlation coefficient between socio-demographic variables, feature dependence scores w.r.t. COVID-19 incidence and RMSE. The displayed variables are limited only to significant correlations with $p < 0.05$

Figure 10 depicts the Spearman rank correlation between the socio-demographic variables of each health area and the dependence scores of each feature capturing the association strength with COVID-19 incidence. Additionally, it displays the correlation of the same socio-demographic variables with the RMSE computed in Sect. 4.3. To aid interpretation, a correlation matrix for all socio-demographic variables can be found in Appendix Sect. A.

Numerous observations can be made (their interpretations follow in Sect. 5). The dependence between total mobility and COVID-19 incidence was negatively correlated with literacy rate. The same can be observed for outgoing mobility and COVID-19 incidence with school enrolment rate as an additional covariate. That is, the higher the literacy and school enrolment rate, the lower the dependence score. Regarding the dependence of outgoing trips and COVID-19 incidence, we observed a positive correlation with the household size and sex ratio along with a negative correlation with the share of the white population and the SDI. In other words, the dependence between the number of outgoing trips and COVID-19 was higher in areas with larger household sizes and a higher proportion of males. The dependence score of the topic *Reflections on Birthdays and New Beginnings* exhibited a high correlation with most socio-demographic variables, with a positive coefficient for the share of multi-ethnic, Asian and black populations, sex ratio and household size and a negative coefficient for the fraction of white people among the population, SDI, age, household income and population density. A similar pattern can be observed for the topic *Casual Conversations and Inside Jokes*. Another notable observation concerns the share of Indigenous people in the population. The association between the normalised fraction of angry/sad posts and COVID-19 incidence was positively correlated with the share of the Indigenous population. It was also the only covariate for which there was a correlation with dependence scores regarding *Health Struggles: Pain, Hunger, and Emotion* and the total number of posts. Additionally, there was a positive correlation between the proportion of Indigenous people with RMSE when using the city-wide selected subset of features. The only other socio-demographic variables correlated with model error were the total population and the number of households which were also highly correlated with each other as depicted in Appendix A.

## Discussion

Our discussion focuses on (1) the results of our experiments and (2) the methodology and its limitations.

### Discussion of the results

Our analysis showed that the features associated most strongly with COVID-19 incidence differed greatly across different health areas. Consequently, varying influential features could be identified per health area, not only allowing for more transparent model development but also enabling an interpretation of the selected features in a socio-demographic context. Consequently, local feature selection on a sub-city level should be considered for future model development when utilising social sensing data such as mobile phone or social media data in the context of epidemiology. This is in line with the findings of Chinazzi et al. (2020) that disease dynamics are location-dependent.

Overall, tourism-, leisure- and COVID-19-related semantic features derived from social media were the features associated most strongly with COVID-19 incidence and selected most frequently on a health area level. This might be interesting for further investigation and model training. Partly, these findings are in line with previous studies that showed associations between social media postings and viral infection cases (Shen et al. 2020). However, an association between tourism- and leisure-related posts and COVID-19 has not been observed yet. Mobility-related and emotional features occurred more rarely at the health area scale. On a city scale, the selected feature set was similar but also contained the feature *Avg. travel distance* along with the fraction of angry and sad posts on social media. The association between average travel distance and COVID-19 incidence suggests that this mobility information could hold predictive power to identify future infection outbreaks, though this connection might also be caused by short-term travel restrictions (Silva et al. 2020) and residents changing their travel behaviour based on their risk perception of COVID-19 (Truong and Truong 2021). Notably, the number of health-related social media posts was only weakly associated with COVID-19 incidence and rarely among the top 10 features. A connection between the two occurred more frequently when allowing for negative temporal lags during the feature selection, i.e., shifting the time series into the past. This suggests that the number of health-related postings only changed *after* COVID-19 incidence rose or fell.

When training prediction models at the health area level, our proposed local feature selection method outperformed traditional methods such as *k*-best MI, LASSO (which only accounts for linear interactions), RFE and FOCI regarding the RMSE, $\sigma _{\text {RMSE}}$ and MSLE. It also achieved competitive values for MAE, $R^2$, MSLE and MAPE, consistently ranking among the top scores. These results highlight the effectiveness of our approach, which, in contrast to the other feature selection methods, also provides insights into the temporal relations between variables. At the city-wide scale, our feature selection method scored the lowest MAE and MAPE and also ranked favourably for the other metrics. The overall best alternative feature selection method was RFE. However, it is important to note that RFE is a model-based feature selection method, whereas our approach is model-free, offering greater flexibility in model selection. Notably, the error metrics were generally lower and the $R^2$ higher at the city level. This can be attributed to the fact that the time series signals exhibited greater variation at the health area level due to the lower data density. However, city-level models lack the ability to provide sub-city insights. For prediction models trained and evaluated at the health area scale, features selected locally yielded a lower RMSE, MSLE, $\sigma _{\text {RMSE}}$ and higher $R^2$ in contrast to the features selected at the city level which exhibited slightly lower MAE and MAPE. This suggests that the locally selected features led to fewer large errors, less variation in errors across health areas and a better overall model fit. These differences in performance can be explained by varying associations between features and COVID-19 incidence among health areas. The standard deviation of dependence scores across all health areas for the local features reached up to 0.28 with the mean being 0.11, indicating a noticeable spatial dependence of association strength. Health-area-level feature selection effectively captures these spatial variations. The fact that *local* features worked better than *global* features in this context goes along with findings of Qiao et al. (2023) who found that perceived severity and susceptibility of COVID-19 on social media varied greatly across counties in the USA. However, our study supports these findings on a fine-grained urban scale. The model error generally decreased when the maximum number of features was increased in the experiments. To ease the interpretation, we eventually decided to use a maximum of 10 features, though this number can be adapted as needed according to a particular study’s specificities. In general, the presented feature selection procedure allows for the training of a much more interpretable model compared to using all features at once.

The correlations observed in Sect. 4.4 align with the general theme of socio-economic and demographic factors influencing disease dynamics. The positive correlation between the relative Indigenous population and the association of angry/sad posts with COVID-19 incidence suggests increased reactions to the pandemic caused either by this demographic or by third influencing factors. A possible explanation might be that Indigenous people were particularly vulnerable during COVID-19 in Brazil as shown by Dias et al. (2021) due to economic instability leading to food insecurity and limited access to healthcare. Their communal way of life heightened exposure to infection, while isolation and the inability to perform cultural rituals led to emotional distress, increased violence, and substance abuse.

**Fig. 11:**
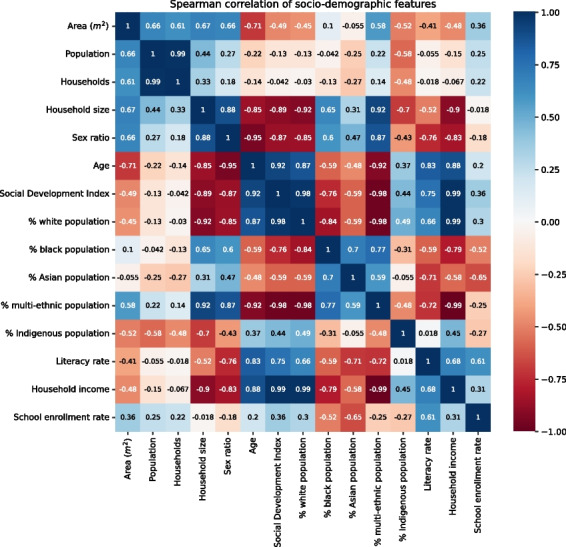
Spearman’s rank correlation for each pair of socio-demographic variables used in the study

Furthermore, the correlations between the dependence of mobility-related variables like the number of trips made on COVID-19 incidence and socio-economic factors like literacy rate, SDI and relative white population suggest that the travel patterns of certain population groups played a more significant role in the spread of COVID-19. As evident by Fig. 11 in the Appendix, the average household income is generally lower in areas with a higher share of black people while (Motte et al. 2016) found that poorer groups have the longest and most difficult commuting journeys, often on crowded buses without air conditioning. It can therefore be assumed that poorer people travelled more and thereby were more involved in spreading the disease. In follow-up research, it may be worth examining these travel patterns more in-depth, explicitly focusing on different sub-groups of the population.

Another notable pattern concerned the high Chatterjee’s Xi values for the topics *Discovering Affordable Hotels and Attractions*, *Celebrating Lives Through Dance and Videos* and COVID-19 incidence. Regarding *Discovering Affordable Hotels and Attractions*, the number of posts, which mostly contained hotel offerings, was zero until late 2020 and then increased, following different patterns relative to COVID-19 incidence depending on the health area. In some areas, the relationship was purely monotonic while the number of offerings dipped after spikes of COVID-19 in other areas. The general rise can be explained by the gradual lift of travel restrictions for international tourists in Brazil (Brito and Rochabrun 2020) while perceived COVID-19 severity and probability of infection simultaneously influences the people’s travel intentions and behaviour (Golets et al. 2023), providing a basis for the general dependence of tourism-related posts on COVID-19 incidence. Posts in the topic *Celebrating Lives Through Dance and Videos* occurred most frequently in the very early stages of the disasters and during times with low COVID-19 incidence. One explanation for this pattern could be the perceived danger and severity of the pandemic, leading to more posts of this kind during perceived low-risk times. This goes in line with reports that the nightlife in Brazil was lively throughout the pandemic even when Carnival was officially cancelled (McCoy 2021).

### Discussion of the methodology

Methodologically, our study provides an approach for discovering associations in mobility, social media posting behaviour, cases of COVID-19 and socio-demographic variables in Rio de Janeiro. It is easily transferable to arbitrary infectious diseases and regions of the world – as long as there is a sufficiently high number of social media posts or mobile phone activity. In our experiments, we also applied our methodology for COVID-19 death numbers and Dengue infections in Rio de Janeiro which yielded similar results in terms of interpretability and error magnitude. The number of Dengue infections in the time frame of our study was rarely above five per health area, however, making any type of quantitative analysis fuzzy. Thus, applying and improving the methodology for vector-based viruses remains a task for future research.

We primarily used feature selection to discover associations among the engineered time series features. A natural next step would be the training of a forecasting model to predict future cases locally. Even though we demonstrated the advantage of our proposed method over traditional feature selection approaches for model training, we also observed that the predictive power of mobility and social media data was still limited beyond 1–2 days. Spikes would not be detected in some cases, making forecasts not reliable enough for real-world usage. Prediction quality was better when auxiliary data such as local or global cases were integrated into the model. One direction for future work could thus concern the development of a prediction model that makes efficient use of mobility and social media but also integrates other knowledge to allow for accurate local infection forecasts (e.g. similar to Lucas et al. 2023).

Each of the building blocks of our methodology also comes with limitations. Mobile phone data and social media data can be collected quickly and easily but might under-represent certain groups of people without a phone or internet access such as children or older adults. This introduces potential sampling bias. However, in our case, we assume that all population groups are represented in the data. According to the Brazilian Institute of Geography and Statistics (IBGE), more than 90% of Brazilians aged 18–59 had a mobile phone for personal use in 2023 (IBGE Coordenação de Pesquisas por Amostra de Domicílios 2024). For those aged 60 and older, the figure was 76%, and for those aged 10–13, it was 55%. Therefore, mobile phone data is slightly biased against the very young population. In contrast, the IBGE found that 84% of Brazilians aged 10–13 used the internet within a three-month period in 2023. This figure was consistently above 88% for all age groups up to 59 years. Among those aged 60 and older, only 66% used the internet within the specified timeframe. Thus, social media data might slightly under-represent the older population. As noted in Sect. 3.1, the mobile phone data utilised in this study only represents an estimated 45% of the population of the municipality of Rio de Janeiro as it was collected by a single telecommunications company. The case is also severe for the social media data: Only 23% of the Brazilian population used the platform X/Twitter regularly in 2021 (Carro 2021). In future research, it would therefore make sense to integrate data from other mobile network operators and more social media platforms, although the availability of such data is often limited due to sharing and API restrictions. Additional work could focus on evaluating the socio-demographic characteristics of social media and mobile phone data, allowing for the incorporation of weighting adjustments during further analyses.

Arguably, our approach holds potential pitfalls such as the Modifiable Areal Unit Problem (MAUP) (Nikparvar and Thill 2021). Thus, the aggregation units must be considered carefully. For instance, we also ran our analysis on a neighbourhood and Regiõe Administrativa (RA) level, however, the associated regions were either very small, holding little or no COVID-19 cases or social media posts, or contained enclosed islands to which social media posts could not be uniquely assigned as their geo-reference also intersected the surroundings.

Semantic information extraction posed one of the most significant challenges during our study. We explored everything from keyword filtering to zero-shot classification of sick tweets using Large Language Models (LLMs) (Wang et al. 2023b), few-shot classification (Tunstall et al. 2022), active learning (Lewis and Gale 1994) and several variants of topic modelling (Grootendorst 2022; Pham et al. 2023; Wang et al. 2023a). Eventually, we chose a combination of zero-shot classification and topic modelling as we could integrate both expert knowledge and additional topics computed unsupervised from the model. We further compared two multilingual and a Portuguese embedding model, *k*-means as well as HDBSCAN for clustering and several methods of topic labelling, arriving at our current configuration. Consequently, the exploration of further methods for meaningful semantic information extraction in space and over time presents a promising research topic with many meaningful applications.

There are also plenty of opportunities for further optimisation of feature selection and model evaluation. We did evaluate other measures of dependence when developing our methodology, specifically (1) the Pearson correlation coefficient which only measures linear dependence (Pearson 1895), (2) MI and Normalised Mutual Information (NMI) which can be estimated for continuous random variables using a *k*-Nearest Neighbour (kNN) estimation (Kraskov et al. 2004; Nagel et al. 2024), though, it is sensitive to the number of neighbours as a parameter and was partly unstable when the number of distinct data points was low and (3) distance correlation (Székely et al. 2007) which is based on pairwise distances but more costly to compute when compared to Chatterjee’s Xi while showing similar performance. For these reasons, we eventually used Chatterjee’s Xi. During an experimental evaluation, MI, NMI and distance correlation yielded similar results compared to Chatterjee’s Xi in terms of the returned features. For the Pearson correlation, the returned feature sets differed more significantly. Going further, those measures of dependence could be evaluated more systematically for feature selection of time series data, specifically focusing on the predictive power of the returned features. Additionally, different prediction models beyond tree-based methods might be explored in contrast to our limited model evaluation.

In general, our study design might be susceptible to spurious correlations. Due to the complexity of the COVID-19 pandemic, misleading associations can occur even for statistically robust measures of non-linear dependence like Chatterjee’s Xi. Results therefore must be interpreted with care and critically reflected with qualitative context in mind. Despite these challenges, this study successfully provides a methodology for the systematic assessment of associations between mobility patterns from mobile phone data, social media postings and COVID-19 infection rates, resulting in insights about human dynamics and knowledge of important variables for model development.

Even though our application regarding COVID-19 in the municipality of Rio de Janeiro is highly specific, we expect our methodology to generalise well beyond the scope of this study. The presented method of feature extraction from mobile phone and social media data is applicable to arbitrary data sets and not restricted to the domain of epidemiology or specific urban regions. The utilised approach for extracting semantic topics from short textual documents has been evaluated on a wide range of data sets including other domains like political social media posts (Grootendorst 2022). Likewise, the emotion classification model used in our workflow was also evaluated on a diverse range of tweet data sets (Bianchi et al. 2022). Consequently, analogous time series features can be extracted for any region of the world. Additionally, our proposed feature method approach can be applied to any time series data beyond our study data. Given that Chatterjee’s Xi is a general measure of dependence, and based on our model evaluation results, we expect our algorithm to perform competitively with other time series data. However, a comprehensive evaluation across various domains remains a task for future research. The full study workflow can be applied to any urban region, provided there is sufficient data and a suitable target time series such as COVID-19 incidence, allowing for effective feature extraction and ranking for a better understanding of urban human dynamics. However, evaluating our approach at different geographic scales (e.g., county-level or country-level) and for varying time-frames remains subject to follow-up research as these investigations fall outside the scope of our study due to the lack of necessary mobile phone and social media data.

## Conclusion

This paper presented a methodology for the assessment of time series features derived from mobile phone OD data and geo-referenced social media posts regarding their association with COVID-19 incidence rates using Chatterjee’s Xi measure of dependence in a feature selection context. We derived 12 mobility time series from a collection of daily OD mobility matrices from 06-04-2020 and 31-08-2021. In addition, we utilised a zero-shot topic modelling approach to obtain 39 time series containing the daily number of social media posts within 19 semantic topics within the same time frame. Furthermore, we used emotion classification to obtain 8 time series capturing the emotions associated with posts. The extracted time series were then used as input to a greedy feature selection algorithm based on Chatterjee’s Xi, enabling us to identify and rank the associations between mobility patterns, social media postings and COVID-19 infection rates in the municipality of Rio de Janeiro, thereby addressing **RQ1**.

The workflow was applied at two spatial scales: On a local health area level and city-wide. Across health areas, tourism-, leisure- and COVID-19-related semantic times series derived from social media were present most frequently among the features selected by our algorithm. That is, they were associated most strongly with COVID-19 incidence. Mobility and emotion time series, on the other hand, rarely made it into the feature sets. At the city level, the selected features exhibited greater diversity in their modality. In addition to semantic time series similar to those at the health area level, the number of posts expressing anger or sadness and the average travel distance were also associated most strongly with COVID-19 incidence. These scale differences provide an answer to **RQ2**. We further found that locally selected features resulted in a lower RMSE, reduced error variation across health areas and a better model fit compared to features selected at a city-wide scale when training prediction models at the health area level. Hence, our findings emphasise the importance of sub-city urban epidemiological analyses, while also hinting towards possible modelling difficulties arising from finer spatial granularity, particularly an increased risk of overfitting and data sparsity. Our feature selection approach additionally yielded better or equivalent model performance compared to traditional feature selection methods, underscoring its effectiveness.

Lastly, socio-demographic variables such as household income, ethnic distribution or education correlated with the dependence scores of multiple features capturing mobility and posting behaviour concerning COVID-19 incidence. These results address **RQ3** and indicate that the strength of associations is influenced by socio-demographic factors. Contextual socio-demographic information, along with mobile phone and social media data, therefore holds significant value for understanding and predicting human dynamics during pandemics of viral infections.

In the broader context of GeoAI research, this study demonstrates how GeoAI methods can be combined with feature selection in urban health contexts, providing insights into the interplay between human mobility, digital social behaviour and public health. We specifically present a framework for identifying and ranking spatiotemporal features derived from human sensors, providing a basis for more explainable feature selection and model development in urban and health studies.

## Data Availability

The raw data supporting the conclusions of this article will be made available by the authors on request.

## Appendix A Socio-demographic correlation matrix

To provide more context and aid the interpretation of the results, a correlation matrix of all socio-demographic variables is depicted in Fig. 11.

## Abbreviations

AP: Area Program´atica da Sa´ude
API: Application Programming Interface
CDR: Call Detail Record
CFS: City-wide Feature Selection
COVID-19: COrona VIrus Disease 2019
FOCI: Feature Ordering by Conditional Independence
GeoAI: Geospatial Artificial Intelligence
GPS: Global Positioning System
HDBSCAN: Hierarchical Density-Based Spatial Clustering of Applications with Noise
IBGE: Brazilian Institute of Geography and Statistics
kNN: k-Nearest Neighbour
LASSO: Least Absolute Shrinkage and Selection Operator
LDA: Latent Dirichlet Allocation
LFS: Local Feature Selection
LIME: Local Interpretable Model-agnostic Explanations
LLM: Large Language Model
MAE: Mean Absolute Error
MAPE: Mean Absolute Percentage Error
MAUP: Modifiable Areal Unit Problem
MI: Mutual Information
ML: Machine Learning
MSLE: Mean Squared Logarithmic Error
NMI: Normalised Mutual Information
NPI: Non-Pharmaceutical Intervention
OD: Origin-Destination
POI: Point of Interest
RA: Regi˜oe Administrativa
RFE: Recursive Feature Elimination
RMSE: Root Mean Square Error
SARS-CoV-2: Severe Acute Respiratory Syndrome CoronaVirus 2
SDI: Social Development Index
SHAP: SHapley Additive exPlanations
UMAP: Uniform Manifold Approximation and Projection
USA: United States of America
WHO: World Health Organisation
XAI: eXplainable Artificial Intelligence
XGBoost: eXtreme Gradient Boosting

## Code availability

The Python code for the methodology of this work will be made available by the authors on request.
